# Supplementary material for: Ferroptosis response segregates small cell lung cancer (SCLC) neuroendocrine subtypes
Source: Nat Commun. 2021 Apr 6;12:2048. doi: 10.1038/s41467-021-22336-4 (PMC8024350; doi:10.1038/s41467-021-22336-4)
Supplement: Supplementary file 1 — Supplementary Information [file 41467_2021_22336_MOESM1_ESM.pdf]

Supplementary Information

Ferroptosis response segregates small cell lung cancer (SCLC) neuroendocrine subtypes

Bebber et al.

Supplementary Figure 1

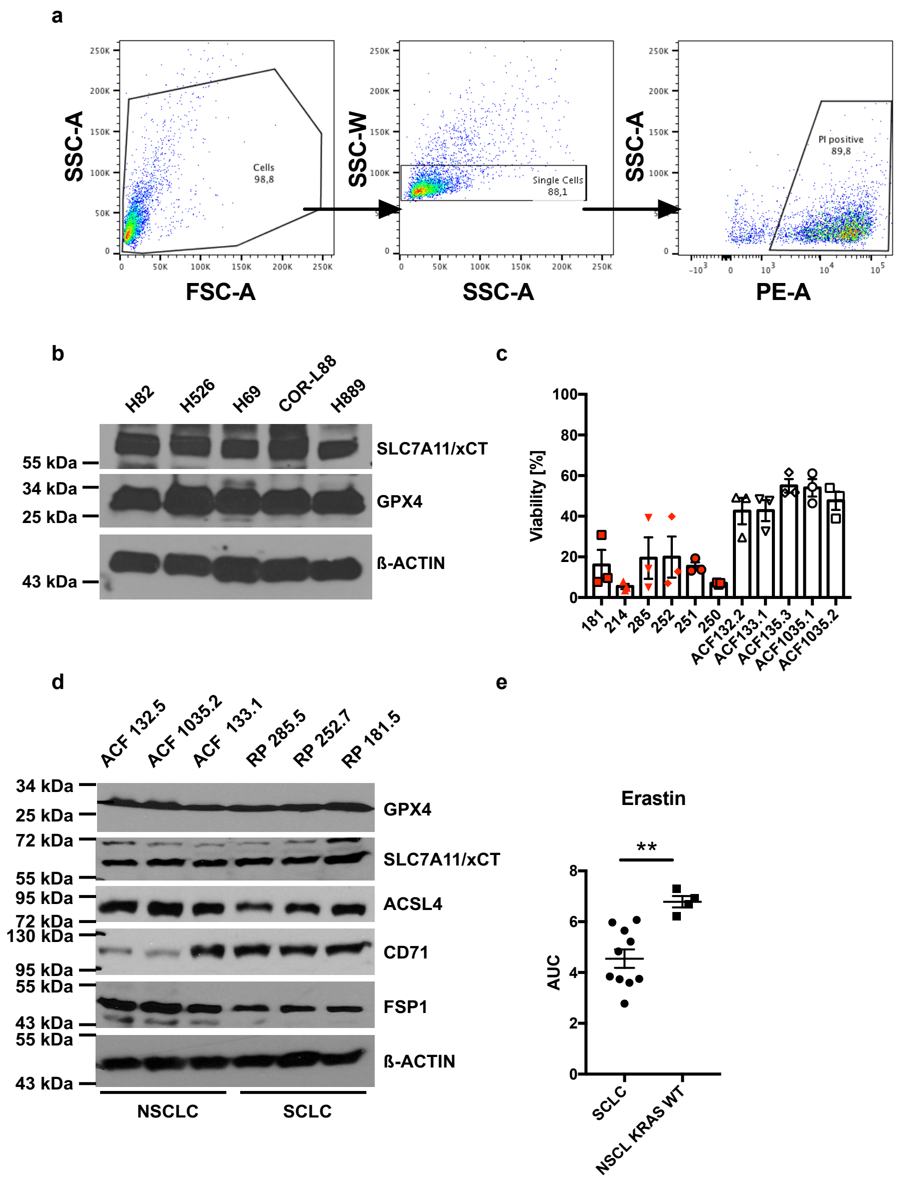

Supplementary Figure 1: SCLC is more prone to respond to erastin than NSCLC

(**a**) Flow cytometry gating strategy to quantify % propidium Iodide (PI) positive cells. Representative plots out of three independent experiments are shown.

(**b**) Western blot of ferroptosis pathway component expression in representative human SCLC cell lines indicated. Representative Western blots are shown.

(**c**) the indicated murine SCLC (red; n=6) and NSCLC (white; n=5) cell lines were treated with erastin [3.33 µM] for 24 h, cell viability was determined by Cell Titer Blue.

(**d**) Western blot of ferroptosis pathway component expression in representative three murine SCLC cell lines (RP) and three murine NSCLC (ACF). Representative Western blots are shown.

(**e**) erastin-treated human SCLC (n=10) and KRAS wild type NSCLC cells (n=4) were plotted for area under the curve (AUC). Source data can be found in Yang et al.[28]. Data are means +/- SEM of three or more independent experiments for each cell line wherever not indicated otherwise. Representative blots are shown. FSC-H, forward scatter-heights, SSC-A, side scatter-area, SSC-W, side scatter-width. Two-tailed unpaired *t* test, ** $p<0.01$. Source Data are provided as Source Data file.

Supplementary Figure 2

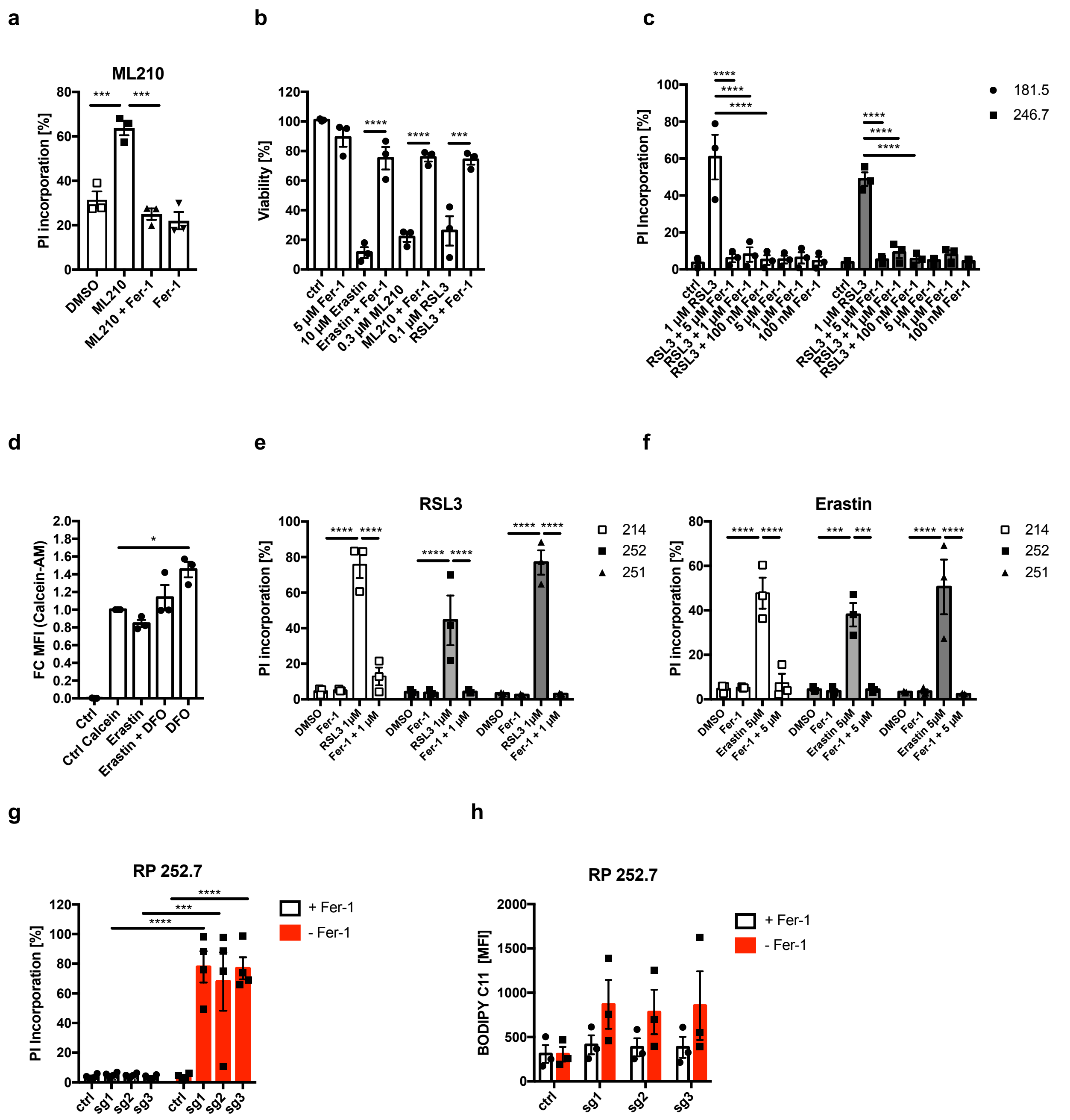

Supplementary Figure 2: **SCLC is sensitive to induction of ferroptosis**

(**a**) Human H82 cells were treated with ML210 [1 µM] +/- Fer-1 for 24 h. Cell death was determined by propidium iodide (PI) uptake and flow cytometry, n=3 biological replicates.

(**b**) Human H82 cells were treated as indicated for 24 h. Cell viability was determined by Cell Titer Blue, n=3 biological replicates.

(**c**) the indicated murine SCLC cells were treated as indicated for 24 h and quantified as in (**a**), n=3 biological replicates.

(d) RP181.5 sticker cells were treated with erastin [10 μM] +/- Deferoxamine (DFO) [100 μM], for 3 h. Labile iron pools were quantified by Calcein-AM staining and flow cytometry. FC (fold change) MFI (mean fluorescence intensity), n=3 biological replicates.

(**e, f**) the indicated murine SCLC cells were treated as indicated +/- Ferrostatin-1 (Fer-1) [5 μM] for 24 h and quantified as in (**a**), n=3 biological replicates.

(g) RP252.7 cells with either control or GPX4-targeting gRNA were grown in the presence of 5 μM Fer-1. Cell death was determined after 24 h of Fer-1 withdrawal by propidium iodide (PI) uptake and flow cytometry, n=3 biological replicates.

(**h**) cells as in (**g**), were cultured in the presence or withdrawal of Fer-1 [5 μM] for 5 h and stained for lipid ROS accumulation using BODIPY C11. Cells were analyzed by flow cytometry and mean fluorescent intensity (MFI) was quantified, n=4 biological replicates. Data are means +/- SEM of three or more independent experiments. One-way ANOVA + Tukey's multiple comparison test, ****$p<0.0001$, *** $p<0.001$, * $p<0.05$. Source Data are provided as Source Data file.

Supplementary Figure 3

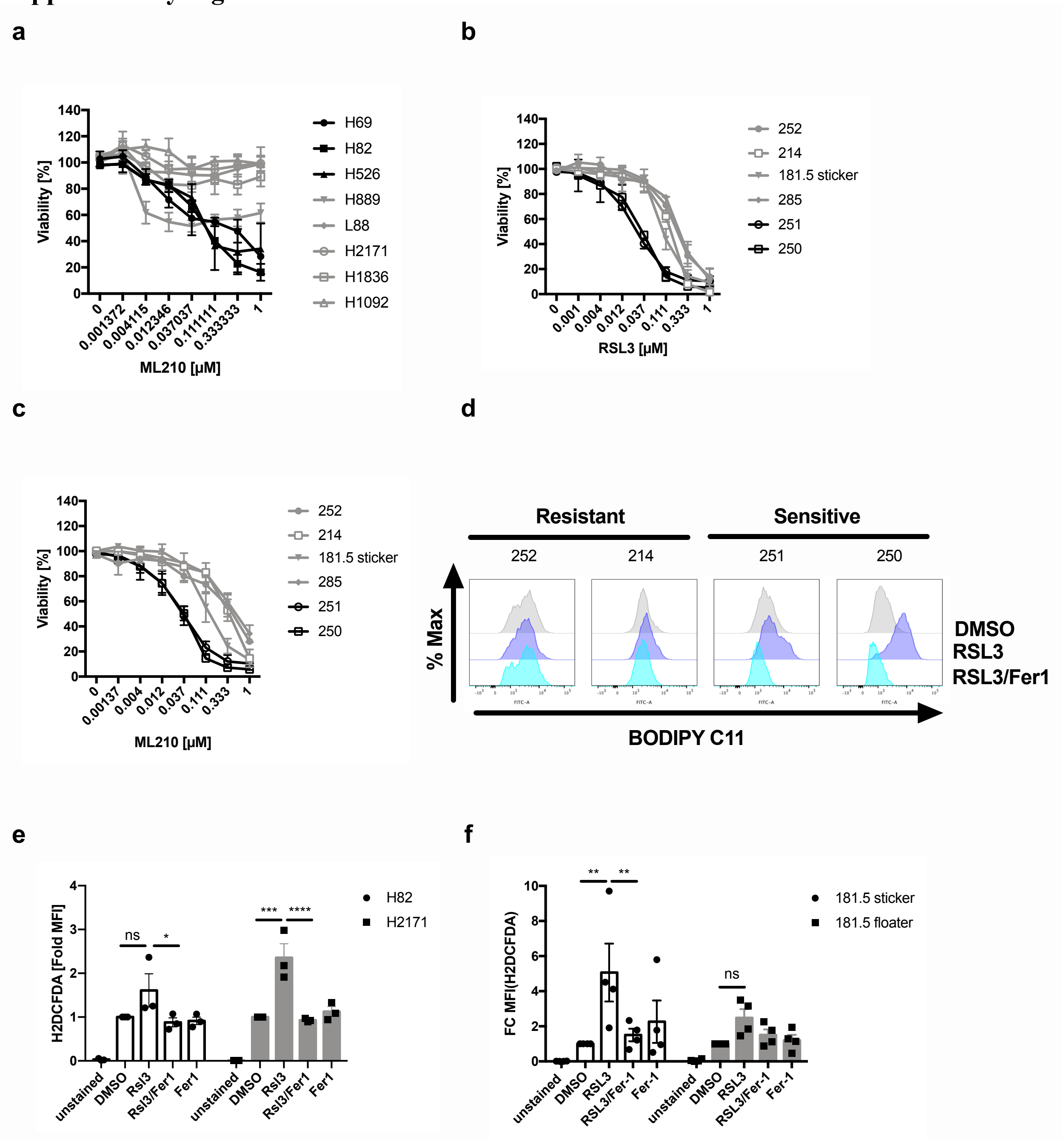

Supplementary Figure 3: SCLC divides into ferroptosis responders and non-responders

(**a**) the indicated eight human SCLC cell lines were treated as indicated for 24 h, cell viability was determined by Cell Titer Blue. For each cell line n=3 biological replicates.

(**b, c**) six murine SCLC cell lines were treated as indicated for 24 h, cell viability was determined by Cell Titer Blue. For each cell line n=3 biological replicates.

(**d**) indicated murine SCLC cells were treated with DMSO, RSL3 [300 nM] +/- Fer-1 [5 μM] for 5 h and stained for lipid ROS accumulation using BODIPY C11. Cells were analyzed by flow cytometry. Grey=DMSO treated, violet=RSL3 treated, turquoise=RSL3/Fer1 treated.

(**e, f**) indicated human (**e**) and murine (**f**) SCLC cells were treated with RSL3 [1μM] for 6 h. Total ROS was determined by H2DCFDA staining. Fold change (FC) mean fluorescence intensity (MFI) was quantified, n=3 biological replicates (**e**), n=4 biological replicates (**f**). Data are means +/- SEM of three or more independent experiments or representative FACS histograms where applicable. One-way ANOVA + Tukey's multiple comparison test, ****p<0.0001, *** p<0.001, ** p<0.01, * p<0.05. Source Data are provided as Source Data file.

Supplementary Figure 4

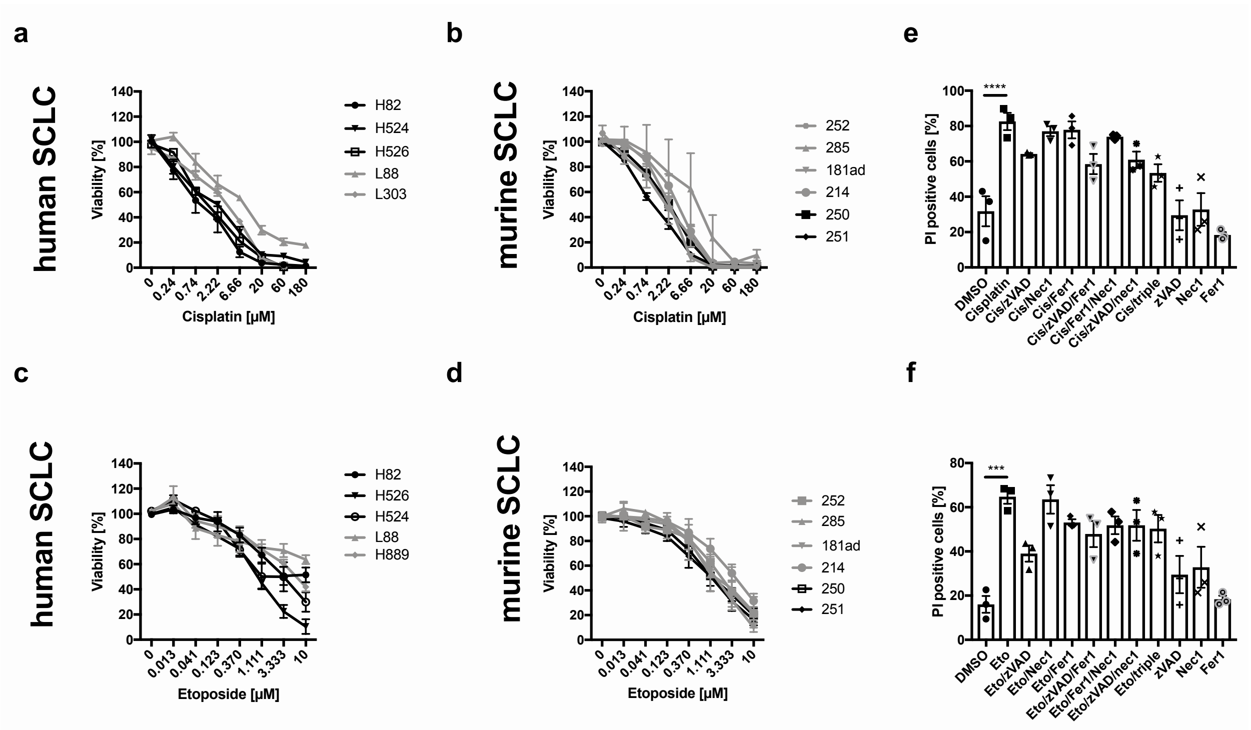

Supplementary Figure 4: Chemotherapy triggers caspase-dependent cell death in SCLC.

(**a**, **c**) the indicated five human SCLC lines were treated with rising concentrations of cisplatin or etoposide for 72 h, cell viability was determined by Cell Titer Blue. For each cell line n=3 biological replicates.

(**b**, **d**) the indicated six murine SCLC lines were treated as indicated for 72 h, cell viability was determined by Cell Titer Blue. For each cell line n=3 biological replicates.

(**e**, **f**) H82 cells were treated with DMSO, cisplatin [180 µM] or etoposide [10 µM] and the indicated combinations using zVAD [20 µM], necrostatin-1 (Nec1) [10 µM] or Ferrostatin-1 (Fer-1) [5 µM] for 72 h. Cell death was determined by propidium iodide (PI) uptake and flow cytometry. triple, zVAD/Nec1/Fer-1, n=3 biological replicates; Data are means +/- SEM of three or more independent experiments. One-way ANOVA + Tukey's multiple comparison, **** p<0.0001, *** p<0.001. Source Data are provided as Source Data file.

Supplementary Figure 5

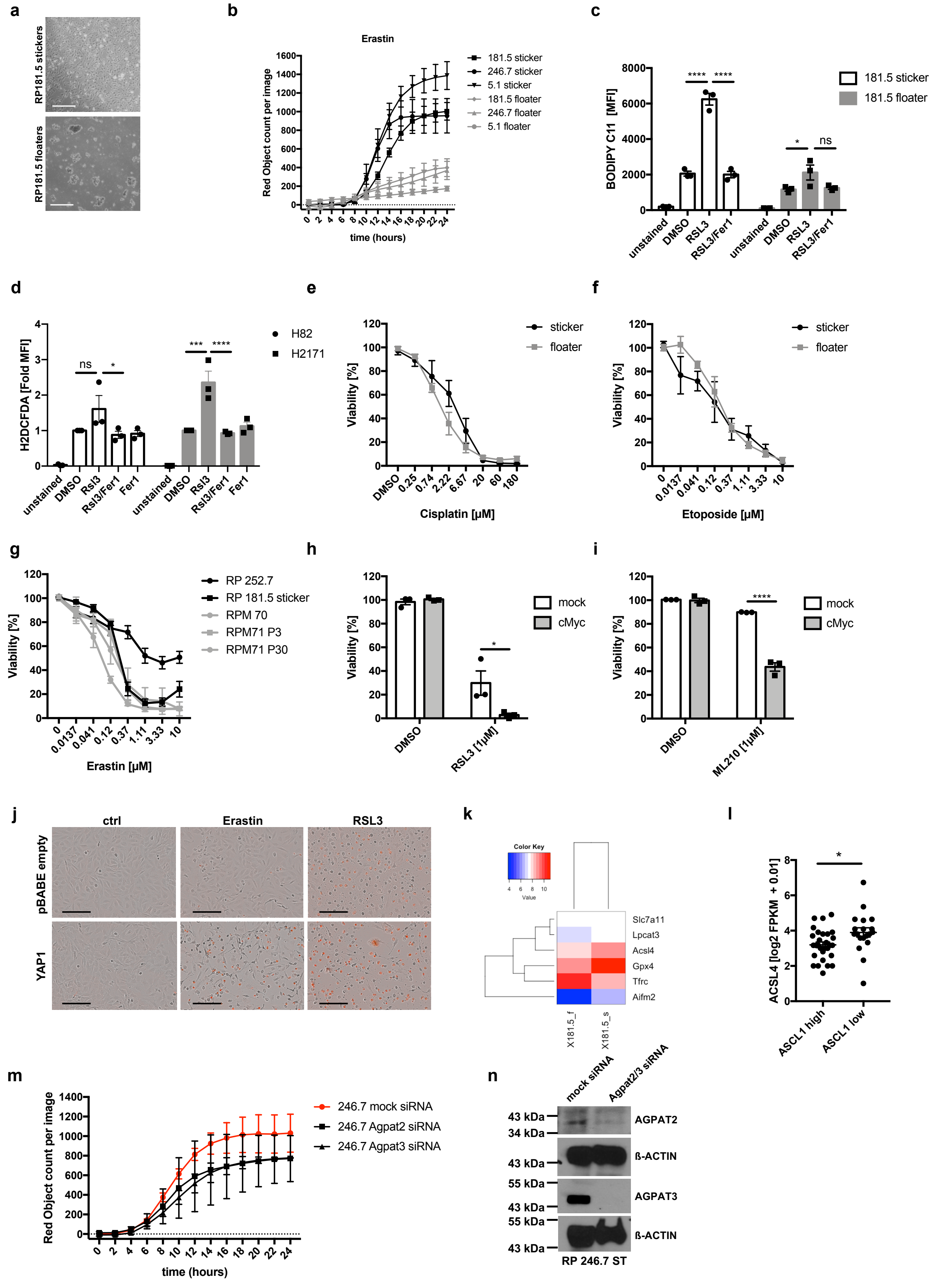

Supplementary Figure 5: non-NE SCLC is more sensitive to ferroptosis and presents with elevated ACSL4 expression

(**a**) representative images of 181.5 stickers and floaters out of at least three independent biological experiments, scale bar=400 µm.

(**b**) The indicated stickers and floater*s* isogenic lines (n=3) were cultured separately and treated with erastin [10 µM] for 24 h in the presence of DRAQ7 [0.1 µM] to visualize dead cells. Images were acquired every 2 h using the IncuCyte S3 bioimaging platform. For each cell line n=3 biological replicates.

(**c**) cells as in (**a**) were treated with DMSO, RSL3 [1 µM] +/- Fer-1 [5 µM] for 5 h and stained for lipid ROS accumulation using BODIPY C11 and analyzed by flow cytometry; mean fluorescent intensity (MFI) was quantified. For each cell line n=3 biological replicates.

(**d**) H82 and H2171 cells were treated with DMSO, RSL3 [1 µM] +/- Fer-1 [5 µM] for 5 h and stained for general ROS accumulation using H2DCFDA and analyzed by flow cytometry; mean fluorescent intensity (MFI) was quantified. For each cell line n=3 biological replicates.

(**e, f**) cells as in (**a**) were treated with rising concentrations of cisplatin or etoposide for 72 h, cell viability was determined by Cell Titer Blue. For each cell line n=3 biological replicates.

(**g**) cells as in (**b**) were treated with rising concentrations of erastin (**f**) for 24 h, cell viability was determined by Cell Titer Blue. For each cell line n=3 biological replicates.

(**h**, **i**) murine RP cells expressing cMyc from the endogenous locus via CRISPRa[37] were treated as indicated for 24 h, cell viability was determined by Cell Titer Blue. For each cell line n=3 biological replicates.

(**j**) 181.5 stickers expressing control or YAP1 5SA-YFP were treated with RSL3 [1 µM] or erastin [10 µM] for 24 h in the presence of DRAQ7 [0.1 µM] to visualize dead cells (red color in image). Images shown are acquired after 10 h using the IncuCyte S3 bioimaging platform. Scale bar = 200 µm. Representative images out of four independent biological replicates are shown.

(**k**) 181.5 stickers and floaters were subjected to RNA-sequencing. FPKM data were log2 transformed (+0.01) and plotted for relative expression of genes involved in ferroptosis. Heatmap color code indicates expression levels between each sample and the average of each gene, dendrogram shows distance between sample populations.

(**l**) the NIH SCLC cell line panel (n=48 cell lines) was segregated by ASCL1 expression (high= log2 (FPKM +0,01)> 3) (low= log2 FPKM +0,01< 3) and ACSL4 mRNA expression is plotted. Cell line panel expression data are available at Expression Atlas https://www.ebi.ac.uk/gxa/home.

(**m**) The indicated murine *sticker* line was subjected to siRNA-mediated knockdown targeting the indicated genes for 72 h. Cells were treated with RSL3 [1 µM] for 24h. Cell death was quantified as in (**b**). For each condition n=3 biological replicates.

(**n**) Representative Western blots of cells as in (**j**) are shown. Data are means +/- SEM of three independent experiments or representative images where applicable. One-way ANOVA + Tukey's multiple comparison test (**c, d, h, i**), two-tailed unpaired *t* test (**l**), ****p<0.0001, * p<0.05. Source Data are provided as Source Data file.

Supplementary Figure 6

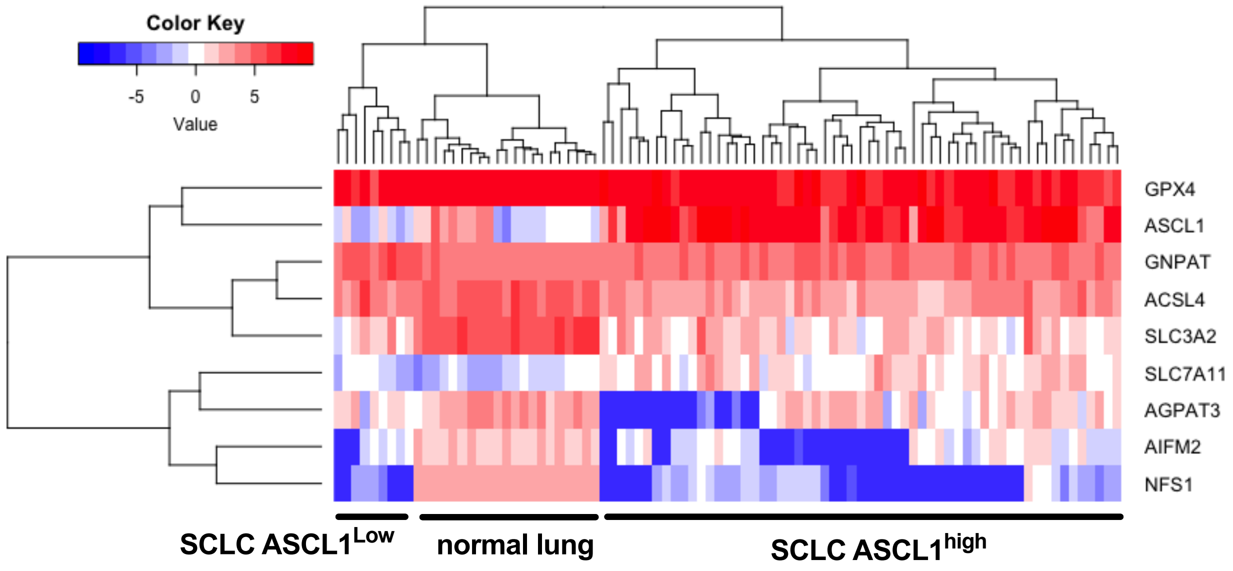

Supplementary Figure 6: ASCL1^{low} SCLC express higher levels of lipid ether metabolism genes

RNA-seq expression data in FPKM (fragments per kilobase of exon model per million reads mapped) from normal lung[84] (n=22) and mostly chemo-naïve SCLC patient samples[7] (n=67) were log2 transformed (+0.01) and plotted for relative expression of the indicated genes. Heatmap color code indicates expression levels between each sample and the average of each gene, dendrogram shows distance between sample populations.

Supplementary Figure 7

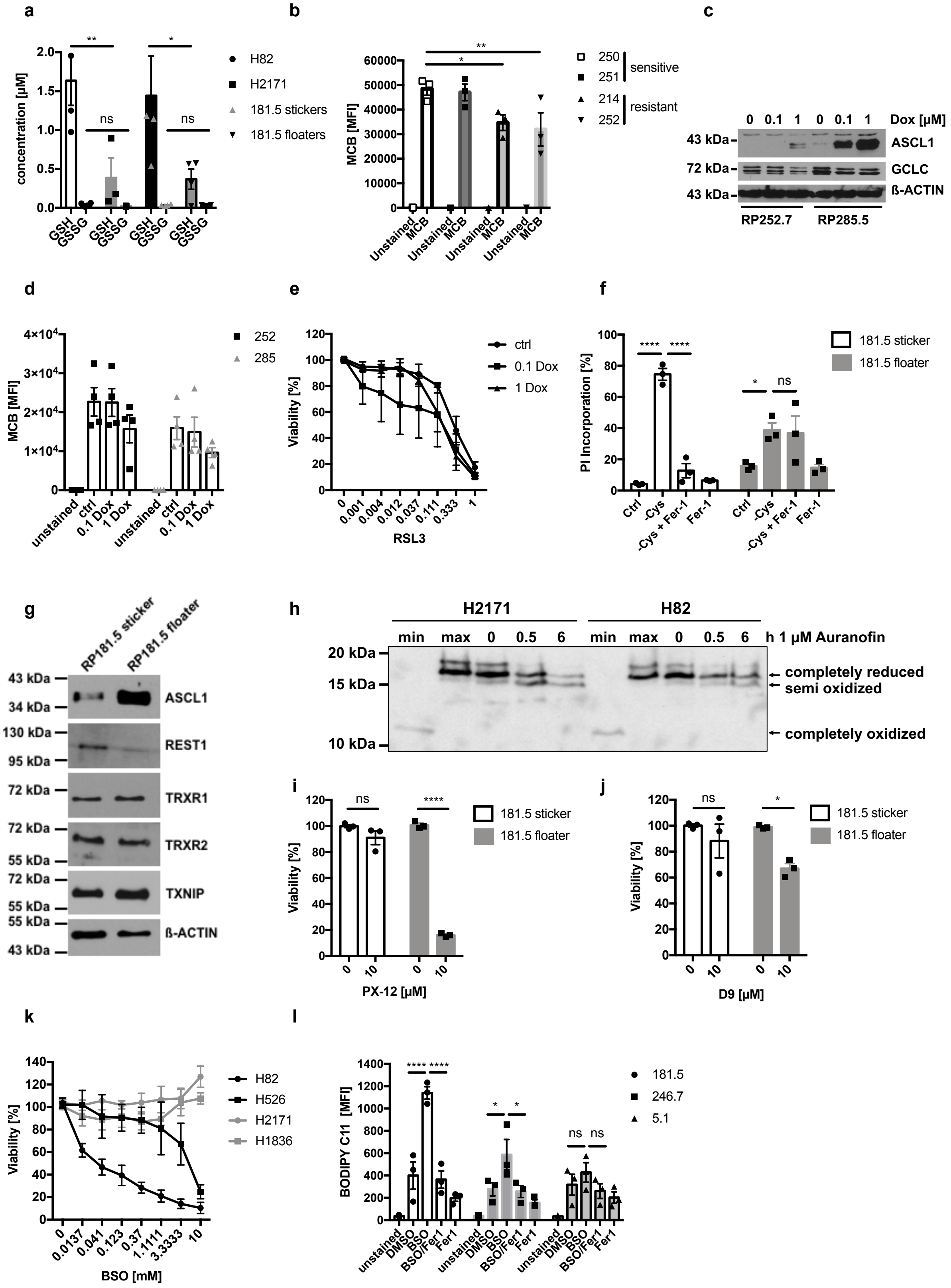

Supplementary Figure 7: NE SCLC suppresses cellular GSH levels and creates dependency on the thioredoxin pathway

(**a**) Cellular GSH concentrations were quantified by GSH/GSSG Glo Assay (Promega) in the indicated cell lines, n(H82, 181.5 stickers, 181.5 floaters)=4, n(H2171)=3 biological replicates.

(**b**) cellular GSH levels were determined by monochlorobimane (MCB) staining and flow cytometry (MFI- mean fluorescent intensity) in the indicated murine SCLC cell lines, n=3 biological replicates.

(**c**) the indicated ASCL1-inducible cell lines were treated as indicated for 24 h. Representative Western blots are shown.

(**d**) cellular GSH levels were determined by monochlorobimane (MCB) staining and flow cytometry (MFI- mean fluorescent intensity) in the indicated murine SCLC cell lines upon 24h of doxycycline induction, n=4 biological replicates.

(**e**) RP285.5 cells were pretreated with 0.1 and 1 μM Doxycycline for 48 h and treated with RSL3 as indicated for 24 h, cell viability was determined by Cell Titer Blue, n=3 biological replicates.

(**f**) 181.5 stickers and floaters were cultured in normal (Ctrl) or cystine-free medium (-Cys) +/- Fer-1 [5 μM] for 24 h, cell death was quantified by propidium iodide (PI) uptake and flow cytometry, n=3 biological replicates.

(**g**) 181.5 stickers and floaters were cultured separately and subjected to Western blot analysis. Representative Western blots are shown.

(**h**) $1.5x10^6$ (H82) and $1x10^6$ (H2171) cells were treated with Auranofin [1 μM] for the indicated times, cells were lysed with 8% (w/v) TCA. TRX shift was analyzed by Western blot. Representative Western blots are shown.

(**i, j**) 181.5 stickers and floaters were cultured separately and treated as indicated for 48 h. Cell viability was determined by Cell Titer Blue, n=3 biological replicates.

(**k**) indicated cells were treated with BSO for 24 h, cell viability was determined by Cell Titer Blue, n=3 biological replicates.

(**l**) the indicated stickers were treated with DMSO, BSO [10 mM] +/- Fer-1 [5 μM] for 5 h and stained for lipid ROS accumulation using BODIPY C11 and analyzed by flow cytometry; mean

fluorescent intensity (MFI) was quantified, n=3 biological replicates. Data are means +/- SEM of three or more independent experiments or representative Western blots where applicable. One-way ANOVA + Tukey's multiple comparison test, ****p<0.0001, ** p<0.01, * p<0.05. Source Data are provided as Source Data file

Supplementary Figure 8

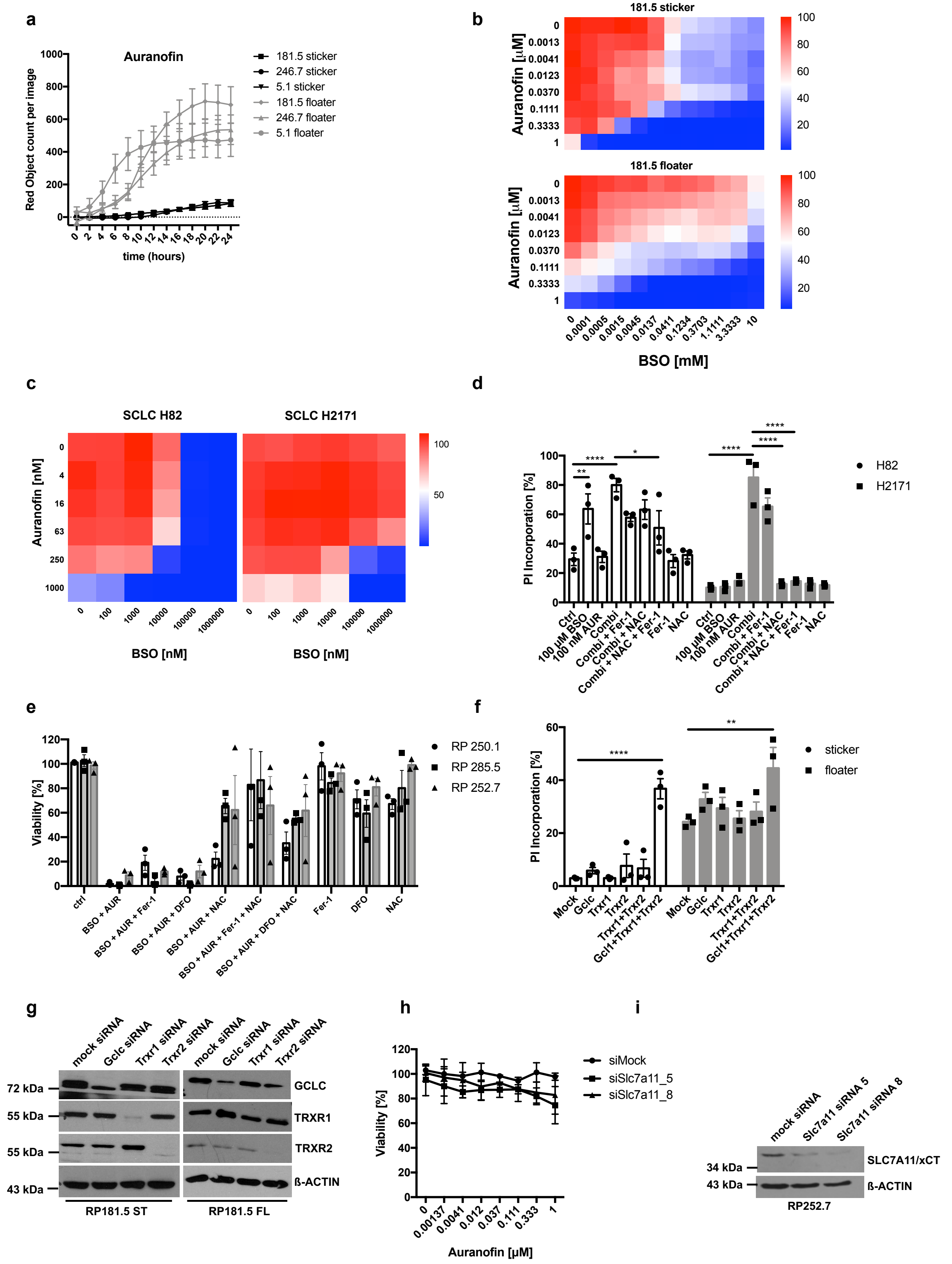

Supplementary Figure 8: BSO/Auranofin-induced cell death is partially ferroptotic and partially dependent on ROS

(**a**) The indicated stickers and floaters isogenic lines (n=3) were cultured separately and treated with Auranofin [1 μM] for 24 h in the presence of DRAQ7 [0.1 μM] to visualize dead cells. Images were acquired every 2 h using the IncuCyte S3 bioimaging platform. For each cell line n=3 biological replicates.

(**b**) 181.5 stickers and floaters were subjected to the indicated cross titrations for 48 h and cell viability was determined by Cell Titer Blue. For each cell line n=3 biological replicates.

(**c**) the indicated human SCLC cell lines were subjected to the cross titrations for 24 h and cell viability was determined by Cell Titer Glow. Heatmap color code indicates viability levels of each sample. For each cell line n=3 biological replicates.

(**d**) the indicated human SCLC cell lines were treated with the indicated combinations of BSO [10 mM], Auranofin (AUR) [1 μM], N-Acetyl Cysteine (NAC) [3 mM], Ferrostatin-1 (Fer-1) [5 μM] for 24 h. Cell death was quantified by propidium iodide (PI) uptake and flow cytometry. For each cell line n=3 biological replicates.

(**e**) the indicated murine SCLC cell lines were either left untreated (ctrl), or treated as indicated: BSO [10 mM], Auranofin (AUR) [1 μM], Deferoxamine (DFO) [100 μM], N-Acetyl Cysteine (NAC) [3 mM], Ferrostatin-1 (Fer-1) [5 μM] for 24 h. Cell viability was determined by Cell Titer Blue. For each cell line n=3 biological replicates.

(**f**) 181.5 stickers and floaters were subjected to siRNA-mediated knockdown targeting the indicated genes for 72 h. Spontaneous cell death was quantified by propidium iodide (PI) uptake and flow cytometry. For each cell line n=3 biological replicates.

(**g**) representative cells as in (**f**) were subjected to Western blot analysis. Representative Western blots are shown.

(**h**) RP252.7 cells were subjected to siRNA-mediated knockdown targeting the indicated gene for 72 h. Cell viability was determined by Cell Titer Blue. For each condition n=3 biological replicates.

(**i**) representative cells as in (**h**) were subjected to Western blot analysis. Representative Western blots are shown. Data are means +/- SEM of three independent experiments or representative FACS plots where applicable. One-way ANOVA + Tukey's multiple comparison test, ****p<0.0001, ** p<0.01, * p<0.05. Source Data are provided as Source Data file.

Supplementary Figure 9

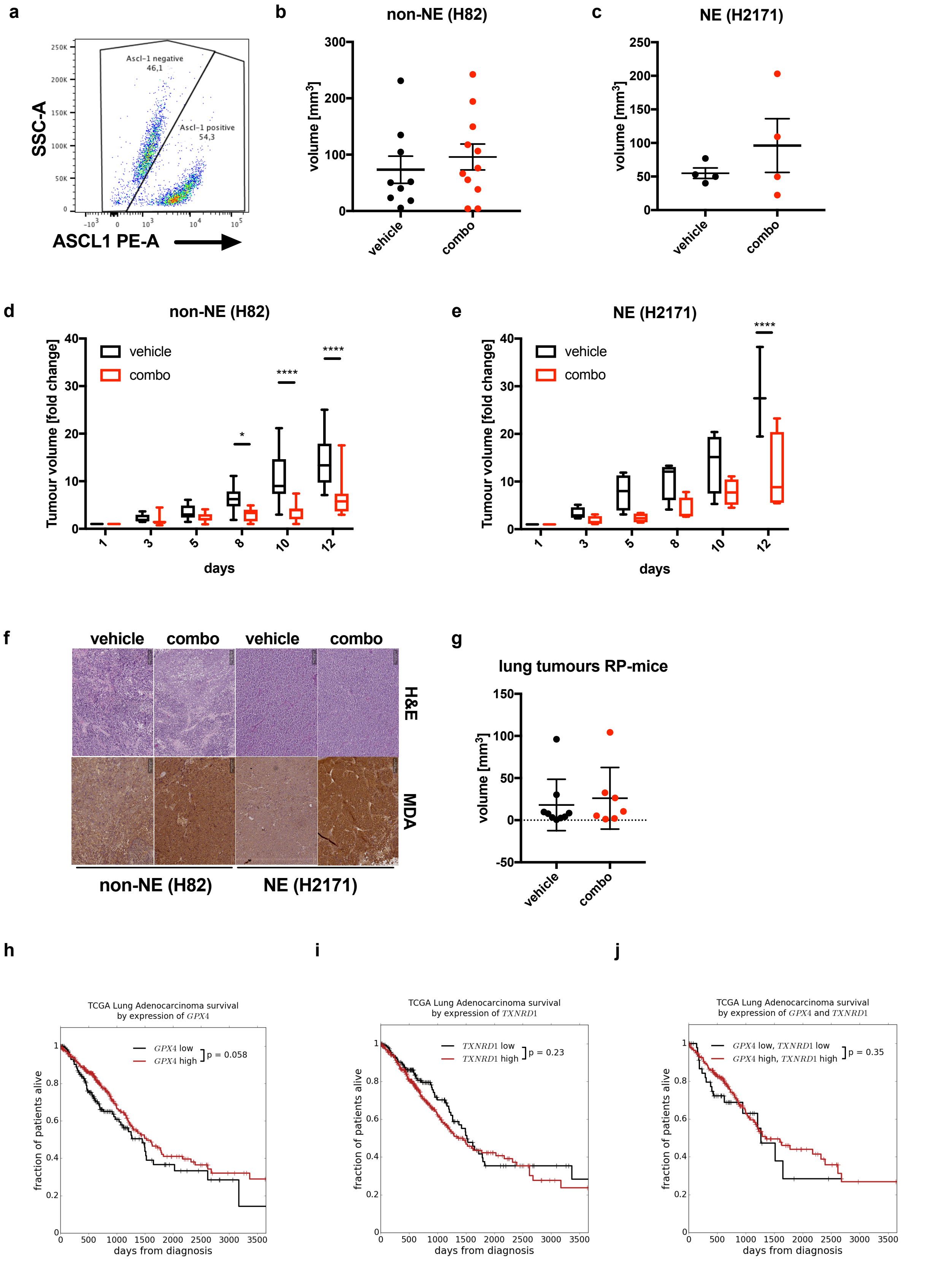

Supplementary Figure 9: BSO/Auranofin is active across SCLC NE subtypes

(**a**) 181.5 stickers and floaters were mixed 50:50 and % of ASCL1+ cells was validated by flow

cytometry before subcutaneous injection.

(**b, c**) 8-weeks old male nude mice were injected with 1.5×10^6 H82 (n=20 tumors) or H2171 (n=8 tumors) cells into flanks. Once measurable, tumors were randomized for the indicated treatment groups, n(H82 vehicle)=9, n(H82 combo)=11, n(H2171 vehicle)=4, n(H2171 combo)=4 biologically independent tumor samples.

(**d**, **e**) 8-weeks old male nude mice were injected with 1.5×10^6 H82 (**d**) and H2171 (**e**) cells into flanks. Once palpable, tumors were treated either with vehicle (H82, n=9; H2171 n=4) or combined BSO [5 mM] in the drinking water and Auranofin 3x per week i.p. [2.5 mg/kg] (H82, n=11, H2171 n=4) for two consecutive weeks. Fold change of initial tumor size is shown. Boxplot center line, mean; box limits, upper and lower quartile; whiskers min. to max.

(**f**) Sections from paraffin-embedded tumors of vehicle or combo-treated mice (**d, e**) were stained by H&E or for MDA. Representative images are shown, scale bar=200 μm.

(**g**) lung tumor volumes were quantified by Horos software using MRI scans. Mice were randomized to obtain two groups with equal mean tumor volume (n=8 for vehicle, n=7 for combo).

(**h**) Kaplan-Meier survival curves for LUAD patients from TCGA (n=503) containing low (low 1/3 n=167, median survival 48.5 months) or high (high 2/3 n=336, median survival 51 months) expression of GPX4 mRNA.

(**i**) as in (**h**), expression of TXNRD1 mRNA was correlated using the same cut-off (low=1/3, median survival 50.5 months; high 2/3, median survival 45.2 months).

(**j**) Kaplan-Meier survival curves for LUAD patients from TCGA with combined low or high GPX4 and TXNRD1 mRNA expression (low/low n=49, median survival 42.2 months; high/high n=218, median survival 45.2 months).

Data are means +/- SEM or representative pictures out of three or more independent experiments or representative FACS plots where applicable. SSC-A, side scatter-area. Significance was tested by two-sided Log-rank (Mantel-Cox) test (**h-j**) or two-way ANOVA + Tukey's multiple comparison test, ****$p<0.0001$, * $p<0.05$. Source Data are provided as Source Data file.

Supplementary Table 1: Primer list

**qPCR**	
Gapdh fw	CTCCCACTCTTCCACCTTCG
Gapdh rv	GCCTCTCTTGCTCAGTGTCC
Agps fw	TACTGTTCGAGGGAGACCGT
Agps rv	CAGCAGCCAGACCACCAAAT
Far1 fw	TAGTGGTCAACACGAGCCTTG
Far1 rv	GGCTTACAGCAATCCAGTAATGA
Lpcat3 fw	ACTGAAGCTAATTGGGCTGTGT
Lpcat3 rv	TCCAGCAATGAAGGGACACC
Acsl4 fw	CTTCCTCTTAAGGCCGGGAC
Acsl4 rv	TGCCATAGCGTTTTTCTTAGATTT
Agpat2 fw	CACCGTGGATAACATGAGCATC
Agpat2 rv	ATTGTCGTTGCGTGTACCCT
Agpat3 fw	AGGAAAACACCTGTCCACGG
Agpat3 rv	ACTGAGAACAGCCGTCCAAG
Gnpat fw	ATGGACGTTCCTAGCTCCTCC
Gnpat rv	CGGGGTGTAGCACTTCATTG
**CRISPR guide RNA**	
Gpx4_sg1_F	CACCG GACGATGCACACGAAACCCC
Gpx4_sg1_R	AAAC GGGGTTTCGTGTGCATCGTC C
Gpx4_sg2_F	CACCG ACGATGCACACGAAACCCCT
Gpx4_sg2_R	AAAC AGGGGTTTCGTGTGCATCGT C
Gpx4_sg3_F	CACCG CGTGTGCATCGTCACCAACG
Gpx4_sg3_R	AAAC CGTTGGTGACGATGCACACG C
**Genotyping**	
p53 1	CAC AAA AAC AGG TTA AAC CCA G
p53 2	AGC ACA TAG GAG GCA GAG AC
RB1 1	CTCATGGACTAGGTTAAGTTGTGG
RB1 2	GCATTTAATTGTCCCCTAATCC
