# Supplementary material for: Ferroptosis response segregates small cell lung cancer (SCLC) neuroendocrine subtypes
Source: Nat Commun. 2021 Apr 6;12:2048. doi: 10.1038/s41467-021-22336-4 (PMC8024350; doi:10.1038/s41467-021-22336-4)
Supplement: Supplementary file 3 — Description of Additional Supplementary Files [file 41467_2021_22336_MOESM3_ESM.pdf]

Description of Additional Supplementary Files

File Name: Supplementary Data 1
Description: 181.5 stickers as compared to 181.5 floaters treated with either DMSO or RSL3 [1 µM] for 5 h and then subjected to lipidomics. Samples for each condition (n=5) were averaged and normalized to the cell number ($2.5x10^6$). Data correspond to one representative out of two independent experiments.
