# Supplementary material for: Ferroptosis response segregates small cell lung cancer (SCLC) neuroendocrine subtypes
Source: Nat Commun. 2021 Apr 6;12:2048. doi: 10.1038/s41467-021-22336-4 (PMC8024350; doi:10.1038/s41467-021-22336-4)
Supplement: Supplementary file 5 — Reporting Summary [file 41467_2021_22336_MOESM5_ESM.pdf]

nature research

Corresponding author(s): Silvia von Karstedt

Last updated by author(s): 02032021

Reporting Summary

Nature Research wishes to improve the reproducibility of the work that we publish. This form provides structure for consistency and transparency in reporting. For further information on Nature Research policies, see Authors & Referees and the Editorial Policy Checklist.

Statistics

For all statistical analyses, confirm that the following items are present in the figure legend, table legend, main text, or Methods section.

n/a	Confirmed	
☐	☒	The exact sample size (*n*) for each experimental group/condition, given as a discrete number and unit of measurement
☐	☒	A statement on whether measurements were taken from distinct samples or whether the same sample was measured repeatedly
☐	☒	The statistical test(s) used AND whether they are one- or two-sided<br>*Only common tests should be described solely by name; describe more complex techniques in the Methods section.*
☒	☐	A description of all covariates tested
☐	☒	A description of any assumptions or corrections, such as tests of normality and adjustment for multiple comparisons
☐	☒	A full description of the statistical parameters including central tendency (e.g. means) or other basic estimates (e.g. regression coefficient) AND variation (e.g. standard deviation) or associated estimates of uncertainty (e.g. confidence intervals)
☐	☒	For null hypothesis testing, the test statistic (e.g. *F*, *t*, *r*) with confidence intervals, effect sizes, degrees of freedom and *P* value noted<br>*Give P values as exact values whenever suitable.*
☒	☐	For Bayesian analysis, information on the choice of priors and Markov chain Monte Carlo settings
☒	☐	For hierarchical and complex designs, identification of the appropriate level for tests and full reporting of outcomes
☒	☐	Estimates of effect sizes (e.g. Cohen's *d*, Pearson's *r*), indicating how they were calculated

Our web collection on statistics for biologists contains articles on many of the points above.

Software and code

Policy information about availability of computer code

Data collection	BD FACS Diva version 8.0, MRI scans were acquired using Philips MR systems Achieva software
Data analysis	Heatmaps visualising cell death pathway component expression were generated using RStudio version 1.1.456 and gplots package version 3.1.1 and RColorBrewer package version 1.1-2 were used. A ranked list of fold differential expression was generated for human cell line RNA seq- data using Excel and analysed by GSEA Desktop v3.0 (https://doi.org/10.1073/pnas.0506580102 and https://doi.org/10.1038/ng1180). FACS data were analysed and quantified using FlowJo 10.4.2. Cell Titer Blue viability assays were analysed using Excel. MRI scans were quantified using Horosv3.3.5. , Lipidomics measurements were analysed by MultiQuant 3.0.2 software (SCIEX). Merged data graphs were plotted using GraphPad Prism 7 for Mac OS X, version 7.0e. Immunofluorescent microscopic images were processed using imageJ, version 1.51 (100).

For manuscripts utilizing custom algorithms or software that are central to the research but not yet described in published literature, software must be made available to editors/reviewers. We strongly encourage code deposition in a community repository (e.g. GitHub). See the Nature Research guidelines for submitting code & software for further information.

Data

Policy information about availability of data

All manuscripts must include a data availability statement. This statement should provide the following information, where applicable:

- Accession codes, unique identifiers, or web links for publicly available datasets
- A list of figures that have associated raw data
- A description of any restrictions on data availability

All data supporting the findings in this study are available from the corresponding author upon reasonable request. The primary data underlying the graphs are provided in the Source Data File. The previously published human SCLC patient (7), normal human lung (84) and human SCLC cell line (36) RNA-seq datasets used in this study are available at the European Genome-phenome Archive, which is hosted by the European Bioinformatics Institute (EBI), under accession code EGAS00001000925 (SCLC patients), EGAS00001000334 (normal lung) and EGAS00001002115 (human SCLC cell lines).

Field-specific reporting

Please select the one below that is the best fit for your research. If you are not sure, read the appropriate sections before making your selection.

☒ Life sciences ☐ Behavioural & social sciences ☐ Ecological, evolutionary & environmental sciences

For a reference copy of the document with all sections, see nature.com/documents/nr-reporting-summary-flat.pdf

Life sciences study design

All studies must disclose on these points even when the disclosure is negative.

Sample size	Sample sizes were not determined a priori. Generally accepted sample sizes were used, with reproducable differences between conditions indicating that the chosen sample sizes were sufficient.
Data exclusions	no data were excluded
Replication	All cell experiments were performed measuring at least duplicates-quadruplicates during each individual experiment and means were calculated. These experiments were repeated independently at least three times to obtain at least three independent means. All figures show means calculated from these three means +/- SEM. All experimental replicates successfully validated the experimental findings.<br>All in vivo mouse experiments were repeated in two independent cohorts with at least three independent biological samples. Again, both independent cohorts successfully replicated the same result.
Randomization	For animal exyperiments, tumour-bearing mice were randomized based on tumour size before treatment. Mice were allocated to experimental groups to obtain an equal mean tumour size and similar distribution of individual sizes before treatment. These randomized tumour sizes prior to treatment are shown in the supplemental information. Other experiments requiring randomization were not performed.
Blinding	xenograft studies, people injecting treatments did not measure tumours. Thereby, measurements were taken in a blinded manner. MRI analysis was done in a similar manner, i.e. MRI measurements were taken by a person other than the one injecting treatments. Histology scoring was done in a blinded manner i.e. person scoring was blinded to treatments the respective animals received. For in vitro cell line work, data collection/analysis was not blinded but repeated by an independent person thereby reducing bias.

Reporting for specific materials, systems and methods

We require information from authors about some types of materials, experimental systems and methods used in many studies. Here, indicate whether each material, system or method listed is relevant to your study. If you are not sure if a list item applies to your research, read the appropriate section before selecting a response.

Materials & experimental systems

n/a	Involved in the study
☐	☒ Antibodies
☐	☒ Eukaryotic cell lines
☒	☐ Palaeontology
☐	☒ Animals and other organisms
☐	☒ Human research participants
☒	☐ Clinical data

Methods

n/a	Involved in the study
☒	☐ ChIP-seq
☐	☒ Flow cytometry
☒	☐ MRI-based neuroimaging

Antibodies

Antibodies used	Unconjugated primary anitbodies: Anti-Gpx4 (Abcam, ab41787, 1:2,000), Anti-xCT (Abcam, ab37185, 1:2,000), Anti-Cas9 (Cell signaling, 14697, 1:1,000,), Anti-ß-actin (Sigma, A1978, 1:10,000), Anti-GCLC (Santa Cruz, sc-166345, 1:1,000), Anti-Ascl1 (BD Pharmingen, 556604, 1:1,000), Anti-Txnrd1 (Cell signaling, 15140S, 1:1,000), Anti-Txnrd2 (Cell signaling, 12029, 1:1,000), Anti-Acsl4 (Santa Cruz Biotechnology, sc-271800, 1:2,000), Anti-Txn1 (Cell signaling, 2429S, 1:1,000), Anti-GAPDH (Cell signaling, 97166S, 1:2,000), Anti-FSP1 (previously described55, kindly provided by M. Conrad, undiluted hybridoma supernatant), Anti-REST1 (ThermoFisher, BS-2590R, 1:1,000), Anti-REST1 (Abcam, ab21635, 1:1,000), Anti-TXNIP (Cell signaling, 14715S, 1:1,000), Anti-CD71 (Santa Cruz, sc-65882, 1:2,000), Anti-cMyc (Abcam, ab32072, 1:2,000), Anti- NCAM (Invitrogen, PA5-79717, 1:1,000), Anti-Vimentin (Abcam, ab137321, 1:1,000), Anti-YAP1 (Cell signaling, #4912, 1:1,000), Anti-Synatophysin (Invitrogen, MA5-14532, 1:1,000), Anti-AGPAT2 (Thermo Fisher, PA5-76010, 1:2,000), Anti-AGPAT3 (Thermo Fisher, PA5-101343, 1:2,000).<br>HRP-conjugated secondary antibodies: goat-anti-mouse-HRP (Linaris GmBH, 20400-1mg, 1:10,000), goat-anti-rabbit-HRP (Linaris GmBH, 20402-1mg, 1:10,000), goat-anti-rat-HRP (Sigma, A9037-1ml, 1:10,000).
Validation	The following antibodies were validated by the supplier:<br><br>Anti-Acsl4 (Santa Cruz Biotechnology, sc-271800, 1:2,000) has been validated by supplier.

Anti-Txn1 (Cell signaling, 2429S, 1:1,000) has been validated by supplier.
Anti-ß-actin (Sigma, A1978, 1:10,000) has been validated by supplier.
Anti-GAPDH (Cell signaling, 97166S, 1:2,000) has been validated by supplier.
Anti-TXNIP (Cell signaling, 14715S, 1:1000) has been validated by supplier.
Anti-REST1 (ThermoFisher, BS-2590R, 1:1000) has been validated by supplier.
Anti-Synaptophysin (Invitrogen, MA5-14532, 1:1000) has been validated by supplier.
Anti-vimentin (Abcam, ab137321, 1:1000) has been validated by supplier.
Anti-FSP1 (was published previosly (Doll et al. 2019 nature, kindly provided by M. Conrad, hybridoma supernatant used undiluted) has been KO validated by supplier (M. Conrad).
Anti-CD71 (Santa Cruz, sc-65882, 1:2,000) has been validated by supplier.
Anti-NCAM (Invitrogen, PA5-79717, 1:1,000) was validated by supplier

The following antibodies were validated by us:
Anti-YAP1 (Cell signaling, #4912, 1:1,000) was validated by us by overexpression of YAP1 5SA
Anti-cMyc (Abcam, ab32072, 1:2,000) was validated by cMyc overexpression
Anti-AGPAT3 (Thermo Fisher, PA5-101343, 1:2000) was validated by us by knockdown
Anti-AGPAT2 (Thermo Fisher, PA5-76010, 1:2000) was validated by us by knockdown
Anti-Gpx4 (Abcam, ab41787, 1:2,000) was validated by CRISPR/Cas9 knockout by us.
Anti-xCT (Abcam, ab37185, 1:2,000) was validated by siRNA-mediated knockdown by us.
Anti-Cas9 (Cell signaling, 14697, 1:1,000,) was validated by overexpression.
Anti-GCLC (Santa Cruz, sc-166345, 1:1,000) was validated by knockdown.
Anti-Ascl1 (BD Pharmingen, 556604, 1:1,000) was validated by knockdown by us (data not shown).
Anti-Txnrd1 (Cell signaling, 15140S, 1:1,000) was validated by knockdown by us (data not shown).
Anti-Txnrd2 (Cell signaling, 12029, 1:1,000) was validated by knockdown by us (data not shown).

Eukaryotic cell lines

Policy information about cell lines

Cell line source(s): NSCLC lines (H460, H727 provided by Prof. Julian Downward and the Francis Crick Institute, London, UK who purchased them from ATCC) MEFs were previsoly generated from mouse embryos and provided by Prof. Manolis Pasparakis. All human SCLC lines were obtained from Prof. Martin Sos who previously puchased them from ATCC

Authentication: All SCLC cell lines where authenticated by STR profiling at Eurofins Genomics.

Mycoplasma contamination: All cell lines were regularly checked for mycoplasma contamination by the mycoplasma tube barcodes service provided by Eurofins Genomics. All cell lines used were confirmed to be mycoplasma negative.

Commonly misidentified lines (See ICLAC register): None of the cell lines used are commonly misidentified lines

Animals and other organisms

Policy information about studies involving animals; ARRIVE guidelines recommended for reporting animal research

Laboratory animals: xenograft studies: male 8-week old NMRI-Foxn1nu/nu mice were used (Janvier)
sticker/floater subcutaneous model: male 8-week old NMRI-Foxn1nu/nu mice were used (Janvier)
RP-SCLC model: male and female 8-12 week old mice were inoculated with adeno-Cre to initiate tumour growth, the mice are on a mixed C57BL/6; 129 background, and were housed in individually ventilated cages (IVCs) at 12h/12H light/dark cycle, 55 +/- 10% humidity and 22 +/- 2°C ambient temperature. Immune-deficient animals received autoclaved food, water and bedding.

Wild animals: no wild animals were used in the study

Field-collected samples: no field-collected samples were used in the study

Ethics oversight: All animal experiments were approved by the local authorities (LANUV, North-Rhine-Westphalia, Germany) and performed under license number 81-02.04.2017.A477. All people involved in animal experiments received prior training and have passed the additionally required personal licensing course (FELASAB).

Note that full information on the approval of the study protocol must also be provided in the manuscript.

Human research participants

Policy information about studies involving human research participants

Population characteristics: Both patients from which CTCs were isolated were diagnosed with stage IV SCLC; Treatment: carboplatin + etoposide, sample received and CTCs enriched at the time of relapse in one case (relapse) and sample received and CTCs enriched at the time of first diagnosis in the other case (naive).

Recruitment: Blood samples from patients with SCLC were obtained either at the time of first diagnosis or at relapse. CTC were isolated and enriched by growth in immune-compromised mice (strain NSG). The success rate of maintaining CTCs in xenograft models is at 20-30%. The factors impacting a successful engraftment are not well understood. However, we and others studied the

	established tumor models which were found to recapitulate the genomic features of the patient's tumors (previously described in Hodgkinson et al (Nature Medicine, 2014) and Drapkin et. al (Cancer Discovery, 2018)).
Ethics oversight	Use of patient material (n=2) and healthy donor PBMCs (n=2) was approved by the institutional ethics committee of the University Hospital of Cologne following written informed consent. We have complied with all relevant ethical regulations pertaining to the use of human patient material.

Note that full information on the approval of the study protocol must also be provided in the manuscript.

Flow Cytometry

Plots

Confirm that:

☒ The axis labels state the marker and fluorochrome used (e.g. CD4-FITC).

☒ The axis scales are clearly visible. Include numbers along axes only for bottom left plot of group (a 'group' is an analysis of identical markers).

☒ All plots are contour plots with outliers or pseudocolor plots.

☒ A numerical value for number of cells or percentage (with statistics) is provided.

Methodology

Sample preparation	Dead cells from human or murine established cell lines were analyzed either from supernatants or adherent cells were detached by Accutase, suspension cells pelleted, washed in PBS and then taken up in FACS buffer (PBS, 2% FCS). For Dead cell stains these were incubated with propidium iodide at 1 microg/ml and subsequently analyzed. For other stains, single cell suspension was incubated with the respective staining antibodies, washed and then analyzed.
Instrument	FACS data were acquired on an LSR Fortessa (cat no. 647788, BS Bioscience), November 2013 model no. 647788E3
Software	Data were acquired using Diva software 8.0 (BD Bioscience), analysis was performed using FlowJo version 10.4.2
Cell population abundance	ASCL1+ cells within all "stickers" were present at about 25%, within all floaters 100% were ASCL1-positive
Gating strategy	In all cases it was sequentially gated on all cells, excluding debris and very large aggregates using FSC-A versus SSC-A gating, then on single cells using SSC-A versus SSC-W, this population was then analyzed for PI fluorescence versus SSC-A to measure dead cells. For all other stainings (BODIPY C11 etc.) it was gated on live cells (PI- or other viability dye-) and this population was measured using the respective fluorescent chanels (BODIPY C11 excitation 520 nm, H2DCFDF and Calcein-AM staining excitation 496 nm, MCB staining 405 nm excitation).

☒ Tick this box to confirm that a figure exemplifying the gating strategy is provided in the Supplementary Information.
